# A spatio-temporal methodology for greenhouse microclimatic mapping

**DOI:** 10.1371/journal.pone.0310454

**Published:** 2024-09-19

**Authors:** Elia Brentarolli, Silvia Locatelli, Carlo Nicoletto, Paolo Sambo, Davide Quaglia, Riccardo Muradore

**Affiliations:** 1 Department of Computer Science, University of Verona, Verona, Italy; 2 DAFNAE Department, University of Padua, Padua, Italy; 3 Department of Engineering for Innovation Medicine (DIMI), University of Verona, Verona, Italy; Chunghwa Telecom Co. Ltd., TAIWAN

## Abstract

Greenhouse internal microclimate has been proven to be non-homogeneous in many aspects. However, this variability is only sometimes considered by greenhouse models, which often calculate climatic variables without any spatial reference. Farmers, on the other hand, may wish to have these differences highlighted as they could lead to aimed actions only for a specific area of the greenhouse, while at the same time, they are not willing to invest in sensors to be installed everywhere. This paper presents a data-driven methodology to generate a virtual 2D map of a greenhouse, which allows farmers to control any critical parameter they desire with minimum investment, as monitoring is done via soft sensing with only a few actual sensors. The proposed flow starts with a set of temporary sensors placed in the points of interest; then, a model for each of them is developed via linear regression and, finally, a map of the entire area can be derived by interpolating values from these models. This allows the generation of accurate models at a reduced cost as temporary sensors can be reused at other locations. The methodology has been tested on adjacent greenhouses and in two farms, where temperature and other climatic variables have been monitored. Experimental results show that the proposed methodology can reach an adjusted *R*^2^ value of 98% for predicting values in different greenhouse locations.

## 1 Introduction

Monitoring climate conditions is a well-known problem in agriculture, as their changes significantly impact plants’ health and growth, even leading to irreversible damages if left uncontrolled [1]. Greenhouses significantly help since farmers can have more control over plants’ microclimate [2]. This has opened new opportunities that researchers have deeply studied, such as finding the best values of climatic variables to either maximize yield [1] or reduce energy consumption [3], water waste [4], and pesticide usage [5]. All these approaches require modeling the climatic behavior of the greenhouse itself to estimate the behavior of the climatic variables and eventually modify it. There are two classes of models. The *mechanistic* models are described by equations derived from physical laws, such as energy or mass conservation. In the first part of their work, Singh *et al*. [6] sum up the history of mechanistic models, shortly describing the most important works since 1970. The *stochastic* models aim to find relationships between given sets of input and output data in order to minimize prediction error by using techniques from Statistics, such as regressions [7], or Artificial Intelligence, such as neural networks [8].

Past works in both fields have assumed the environment to be uniform [9] to reduce model complexity, while climate heterogeneity can be observed inside the greenhouse [10]. These differences in the climate may lead to irregular growth of crops and, if not considered, may hinder plant disease prediction. In fact, a particular disease may appear at a specific point with favorable conditions and spread from there, even if the average climate behavior seems unfavorable. Nowadays, various models can predict the insurgence of specific diseases based on climatic conditions, e.g., in [11]; therefore, a fine-grain vision of the greenhouse microclimate can greatly help detect possible critical points and act preemptively.

To take into account this heterogeneity, different monitoring strategies have been proposed, ranging from sensors scattering as in [12] to Computational Fluid Dynamics (CFD), which links temperature to its physical causes, e.g., sunlight absorption, reflection, and refraction [13, 14]. In [15], authors took a step further and introduced virtual sensors inside the greenhouse via regression techniques using outside data as inputs and then generated a microclimatic model in real-time by using CFD. Such solutions can produce accurate results to describe the greenhouse’s microclimate heterogeneity. However, many sensors (and related infrastructure) are needed in the case of sensor scattering, whereas, in the case of CFD models, hundreds of physical parameters should be specified. Furthermore, farmers are usually not interested in climate variable values for every point of their greenhouse but rather in particular *points of interest*, e.g., in correspondence of plant rows. Finally, not every greenhouse location can host a sensor because of spatial and working constraints.

**Fig 1:**
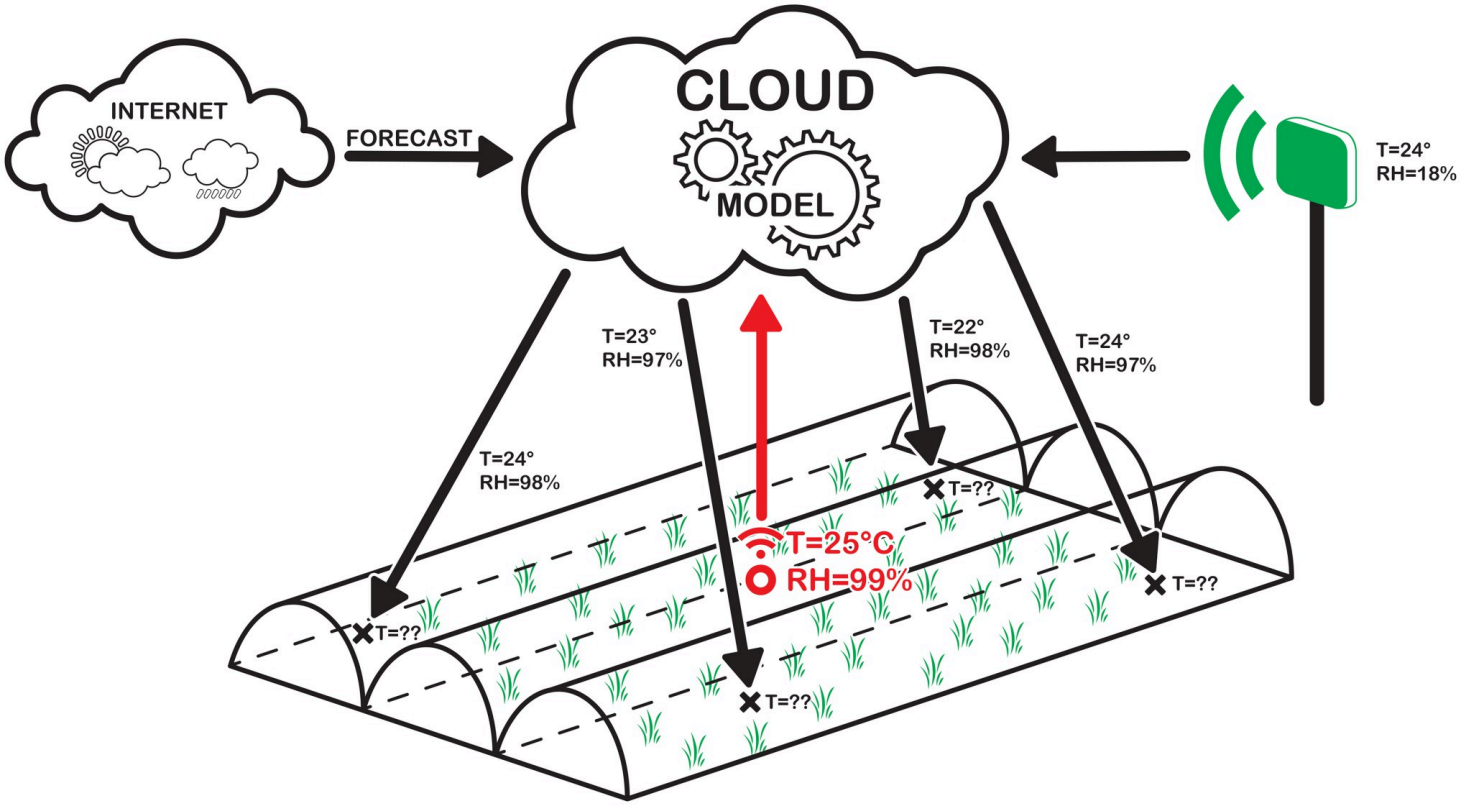
Scheme for the proposed methodology. Proposed methodology for monitoring multiple points of interest based on a single sensor.

In this paper, a new approach is proposed by combining permanent and temporary sensors, external sources of information, and statistical regression to create a microclimatic map of the greenhouse and to extend the concept of *soft sensors* (also called “virtual sensors”) with different categories depending on the modeling technique and their usage. As depicted in Fig 1, the proposed infrastructure mainly consists of the following entities:

1. Persistent sensors, installed permanently in low number to reduce costs and encumbrance, provide accurate data regarding the inside of the greenhouse (red circle in the figure) and its outside (green box in the figure).
2. additional information sources, e.g., for weather forecasting.
3. *Sparse Soft Sensors*; each of them is created by correlating data from persistent sensors to data sampled by a temporary sensor in the same location (black crosses in the figure).
4. A *2D-Sensor* is a soft sensor describing the 2-dimensional behavior of a climatic variable obtained by interpolating data from *Sparse Soft Sensors*.

A more accurate definition of *Sparse Soft Sensors* and *2D-Sensor* will be given in subsection 2.4: Soft Sensors development, while the mathematical procedure to obtain them is provided in subsection 3.2: *Sparse Soft Sensors and Forecast Sparse Sensors* and subsection 3.4: *2D-Sensor* respectively. In the initial setup, only persistent and temporary sensors are placed, accumulating readings in the points of interest. Then, from those data, *Sparse Soft Sensors* can be modeled and the temporary sensors removed. Finally, the *2D-Sensor* is created, covering locations not directly considered in the sampling process. Temporary sensors can be moved to different locations to restart the sampling process, thus increasing the covered area or map accuracy. Additionally, this approach can also take into account weather forecasts and detect anomalous readings of persistent sensors. Weather forecasting is an important aspect to be considered in agriculture since it allows us to act in advance according to future climatic conditions. Our methodology allows us to correlate weather forecasts with actual data read from the field to estimate future climatic conditions at the various points of the greenhouse. Using the same statistical techniques, we also create a model of each persistent sensor to detect possible anomalies that can significantly compromise the global reliability of the monitoring system. We will label models that use forecast data *Forecast Sparse Sensors*, while models for anomalies detection *Anomaly-Detection Soft Sensors*.

Soft sensing is well-known in statistics and traditionally applied to process control, being used as methods to control industrial plants from early ‘90s, as show in [16–18], and generally it focuses on the idea of developing approaches and algorithms to estimate or predict physical quantities in industrial processes based on the available measurements and knowledge, as defined in Jiang *et al*.’s review on the topic [19]. In agriculture, however, soft sensing has been employed only recently. In [20], the authors developed soft sensors to monitor crop transpiration, for which affordable sensors were not available, while in [15] soft sensors were used to run in real-time the CFD model developed in that work. Authors of [21] first created a complete CFD model of the greenhouse whose outputs were then used to train the soft sensors. Vice versa, our approach is purely data-driven, and we train soft sensors directly with data from persistent and temporary sensors. We claim that building a CFD microclimatic model of the greenhouse is more complex than simply considering data from both persistent and temporary sensors.

The main contribution of our paper is a methodology that:

- models greenhouses’ microclimate by using only sensed data and statistical methods;
- can be implemented in a software system that allows farmers to easily create the model in autonomy by just collecting data sensed by the persistent and temporary sensors;
- is scalable since new readings from temporary sensors can be added to progressively increase the accuracy of the microclimate estimation and the coverage of the points of interest;
- can dynamically detect possible failures in persistent sensors, as already pointed out in [22];
- predicts future values of the microclimate variables by using weather forecasts.

This paper is organized as follows: in section 2: Material and methods the experimental setup is described, and regression techniques are recalled; in section 3: Soft Sensors modeling, a hierarchy of soft sensors is defined based on their modeling techniques and usage and for each category its design methodologies are presented; in section 4: Results experimental results are shown while their discussion is given in section 5: Discussion; finally conclusions are drawn in section 6: Conclusion.

## 2 Material and methods

### 2.1 Greenhouse

The research was conducted in greenhouse tunnels of two farms located near Verona (45° 20′ 25.9″N 11° 05′ 12.3″E and 45° 20′ 47.13″N 11° 1′ 46.4″E respectively) in north-eastern Italy. The climate of this area is characterized by warm, humid summers and moderately cold winters. The experiment spanned from late August 2021 to early July 2022.

Each tunnel is 275.6 m^2^ (50 m x 5.3 m) northeast-southwest oriented and covered with transparent plastic film. It represents the module of a multispan greenhouse structure typical of this geographical area. Before crop transplanting, the soil was tilled and subsequently mulched with a semitransparent green polyethylene film. For the duration of the experiment, both farms changed cultivated crops according to the season: the first farm cycled through eggplants, then lettuce, and finally cucumbers; the second one cycled through tomatoes, lettuce and, finally, eggplants.

### 2.2 Networked persistent sensors

**Fig 2:**
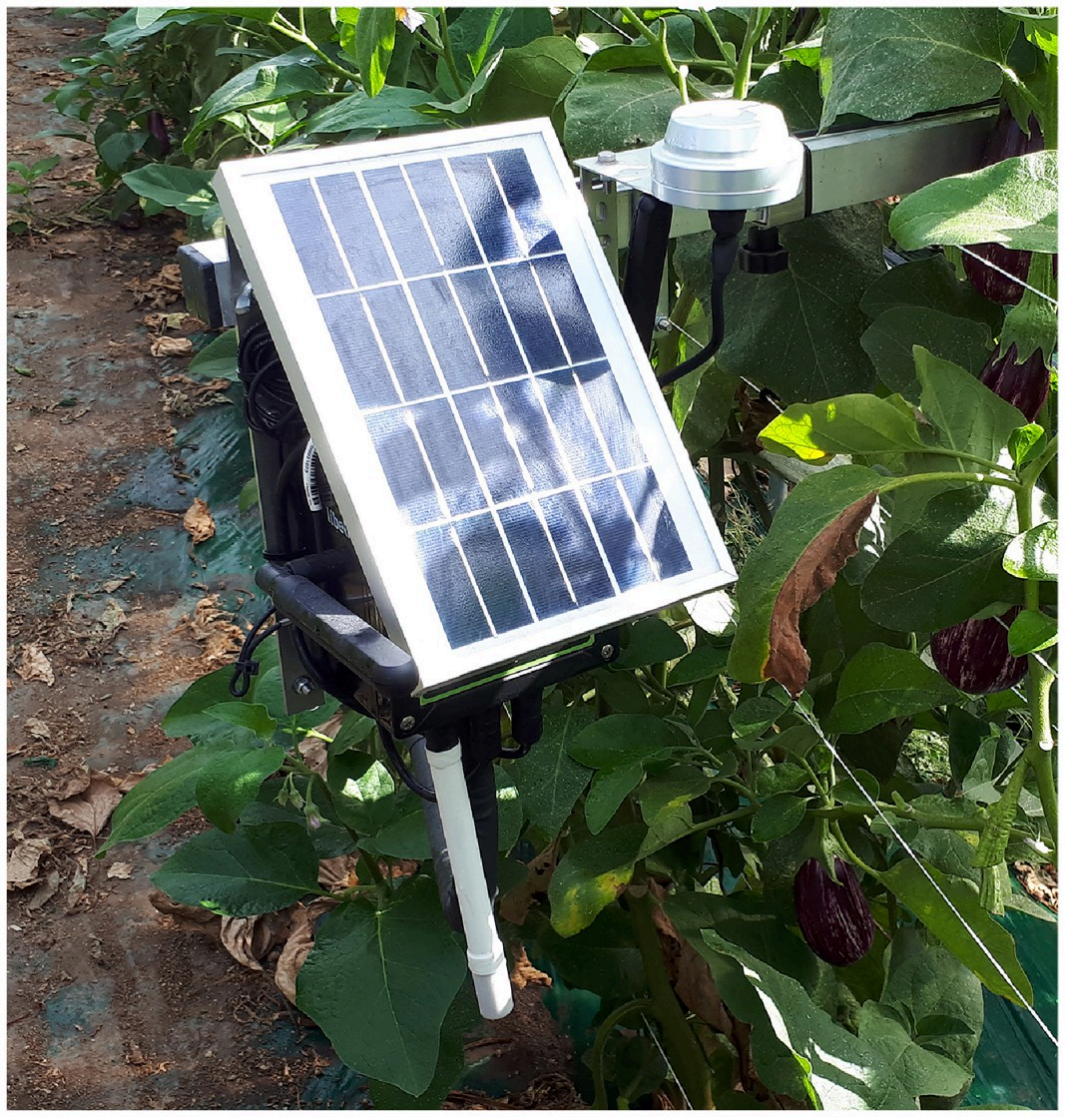
The Evja station. The Evja station hosting persistent sensors.

The primary permanent data sources consist of two microclimatic stations by *Evja s.r.l*. installed in each farm. The stations (Fig 2) have been installed in the greenhouses’ center and attached to telescopic poles, which allow farmers to move them into a position that does not hinder working practices. Each station is equipped with the following sensors: an air temperature (AT) sensor (range of -40 to 85°C), a relative humidity (RH) sensor (range 0 to 100%), a solar radiation sensor (range 0—250 000 lx or 0—316 W m^−2^), a soil humidity sensor (0—80% VWC) and a soil temperature sensor (-40 to 60°C). Furthermore, the stations are also equipped with a leaf wetness sensor, which is not used in this work due to drip irrigation, and a soil electrical conductivity sensor, which is not used as this metric does not play a significant role in temperature estimation. The stations are equipped with a photovoltaic unit to recharge their internal battery. Sampling time was set to fifteen minutes, the minimum interval allowed by the stations; data were stored in the cloud through a SIM-based Internet connection and accessed using MQTT protocol.

**Fig 3:**
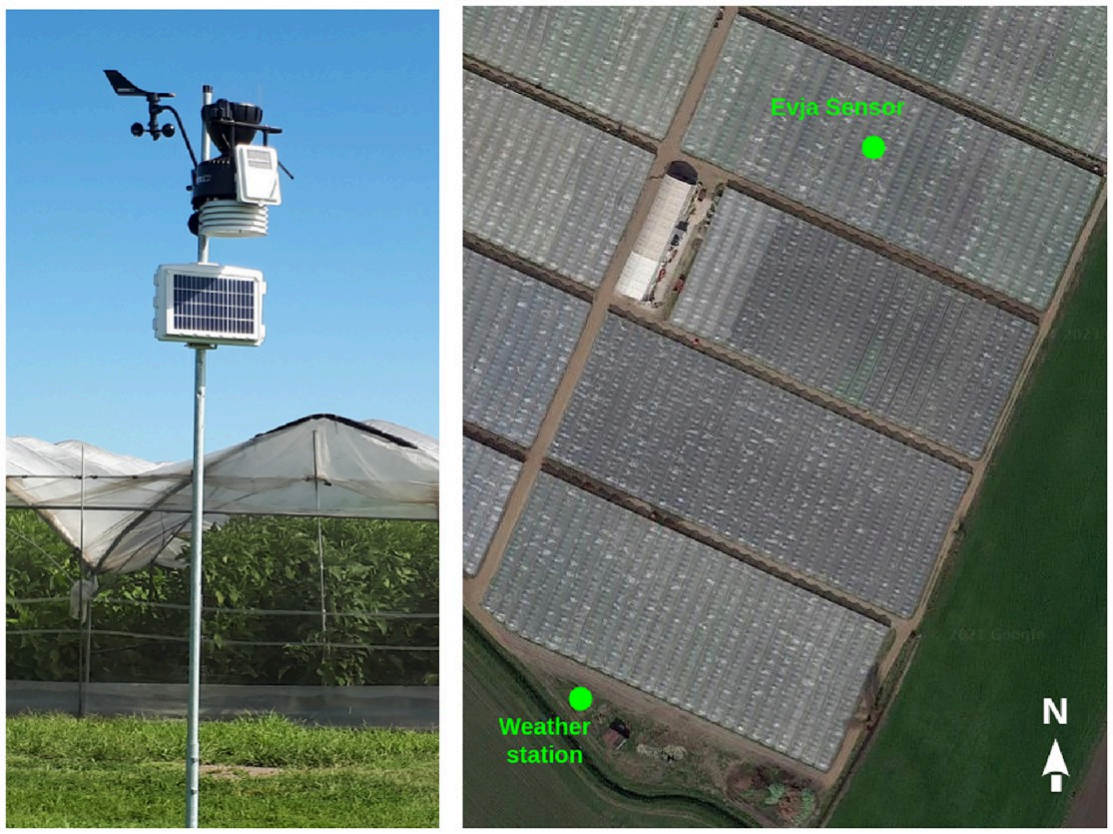
Weather station and its position. Aerial photo of the greenhouse with the positions of the weather station and of the Evja sensors.

A Davis Vantage Pro2 weather station (Fig 3) was also installed relatively close to the greenhouses to give our system a full view of the external climatic conditions. The station is equipped with various sensors (such as barometric pressure, temperature, relative humidity, wind speed, rainfall, and solar radiation) capable of computing dew point, wet bulb temperature, and heat index. This station has a sample time of fifteen minutes and is synchronized with the Evja sensors.

### 2.3 Definition of points of interest with placement of temporary sensors

**Fig 4:**
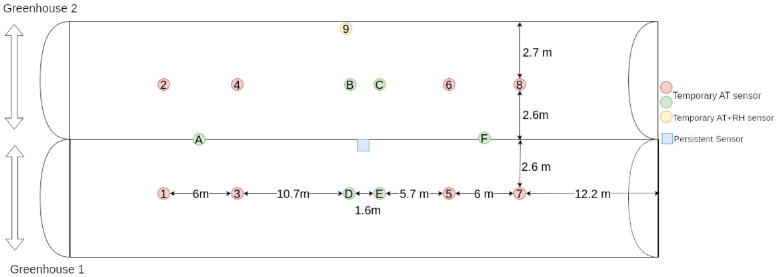
Indoor sensors positioning. Position of the Evja persistent and temporary sensors across the two greenhouses. Sensors denoted by letters were added in a second moment for validation purpose.

A total of nine points of interest between two adjacent greenhouses were chosen for each farm: in positions 1 to 8 HOBO S-TMB-M0xx air temperature sensors have been placed and connected to two UX120–006M loggers providing four channels each; in position 9 a HOBO UX100–011A Data Logger was used to monitor both air temperature and relative humidity. Fig 4 shows the positioning of all sensors.

### 2.4 Soft sensors development

For each point of interest identified in the previous section, a soft sensor has been modeled to monitor the temperature in such a location, using data from temporary sensors as a base. Moreover, besides soft sensors at specific positions, we also modeled a soft sensor capable of computing a temperature map that evolves through time, thus extending the monitored area to previously unmonitored positions. In addition, we also implemented soft sensors capable of using general weather forecasts and localizing them for the points of interest considered. Finally, we computed soft sensors to detect abnormal readings from permanent sensors, identify their faulted state, and temporarily substitute their reading until reparations to guarantee operational continuity.

**Fig 5:**
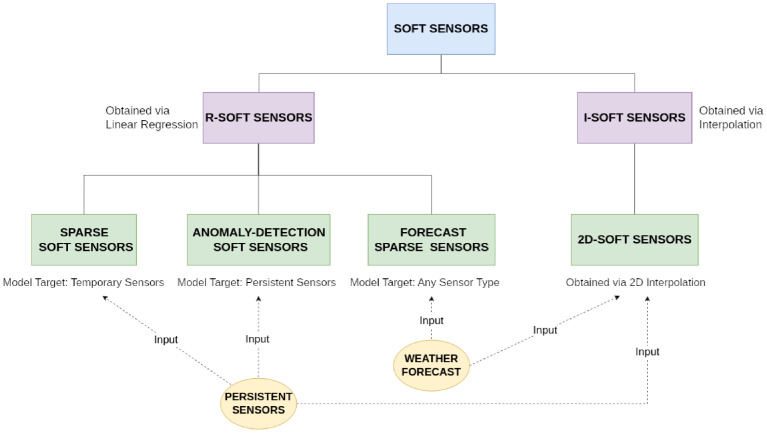
Soft sensor’s hierarchy. Hierarchy of Soft Sensors and their relationships with available data sources.

Since the main differences between different types of soft sensors lie in the mathematical operations used to compute them, a hierarchy was developed as depicted in Fig 5. At the top are two main types of soft sensors, i.e., *Regressed Soft Sensors*, or *R-Soft Sensors*, obtained by using regression techniques with different inputs and outputs, and *Interpolated Soft Sensors*, or *I-Soft Sensors*, which are instead modeled via interpolation. *R-Soft Sensors* can then be divided as: *Sparse Soft Sensors*, which model the current temperature value at specific locations as a function of data provided by the weather station and the microclimatic station; *Forecast Sparse Sensors*, that model future temperature at given positions using general weather forecast downloaded by a web portal; *Anomaly Detection Soft Sensors*, which model the current output of a single sensor of either the microclimatic station or the weather station as a function of data provided by other remaining persistent sensors. On the other hand, *2D-Sensors* are a subtype of *I-Soft Sensors* which computes temperature at any location in the greenhouse through a 2-dimensional interpolation of data provided both by persistent sensors and either *Sparse Soft Sensors*, when requesting current temperature or *Sparse Forecast Sensors*, if future values are needed instead.

In total, our work led to the creation of nine *Sparse Soft Sensors*, nine *Forecast Sparse Sensors*, one for each point of interest identified (and later increased to fifteen each), more than ten *Anomaly Detection Soft Sensors* between the Evja stations and the Davis station to account for all installed sensors, and an unspecified amount of *2D-Sensor*, as with each new set of measures a new sensor has to be computed, regardless of measure coming from *Sparse Soft Sensors* or *Forecast Sparse Sensors*.

### 2.5 Linear regression techniques

Linear regression is a statistical approach to develop a model capable of estimating an output variable value starting from one or more input variables. The model is obtained by finding the linear correlation between historical recordings of such input and output variables.

$$\begin{array}{c} Y=XA+E \end{array}$$ (1)

In Eq 1 $Y=[\begin{array}{cccc} y_{1} & y_{2} & \ldots & y_{n} \end{array}{]}^{T}$ is the output column vector storing values over a window of *n* sample periods and

$$\begin{array}{c} X=[\begin{array}{cccc} x_{1}^{1} & x_{1}^{2} & \cdots & x_{1}^{m}\\ \vdots & \vdots & & \vdots \\ x_{n}^{1} & x_{n}^{2} & \cdots & x_{n}^{m} \end{array}]=[\begin{array}{c} x_{1}\\ \vdots \\ x_{n} \end{array}] \end{array}$$

is an *n* by *m* input matrix where *m* is the number of different input variables *x*_*i*_ and *n* is the temporal sampling window. $A=[\begin{array}{ccc} a_{1} & \ldots & a_{m} \end{array}{]}^{T}$ is the unknown linear estimator to be computed from historical data and *E* is the error vector. Objective of the regression is to find the coefficients *a*_1_, …, *a*_*m*_ that minimize the error vector; by using the least square method we have

$$\begin{array}{c} \hat{A}=[\begin{array}{cccc} {\hat{a}}_{1} & {\hat{a}}_{2} & \ldots & {\hat{a}}_{n} \end{array}]={\left(X^{T}X\right)}^{-1}X^{T}Y \end{array}$$ (2)

where ${\hat{a}}_{i}$ is the predictor of *y*_*i*_ with respect to *x*_*i*_. The matrix $\hat{A}$ can be used to estimate other unknown values $\hat{Y}$ only using the available inputs:

$$\begin{array}{c} \hat{Y}=X\hat{A} \end{array}$$ (3)

The linear regression employs all given inputs to create a model for the output. This means that if unrelated variables are present among the input variables, they will act as disturbances for the regression, reducing its accuracy. For this reason, improved regression methods have been developed to remove the uncorrelated inputs automatically. Here, two of them will be recalled.

The first method is the *lasso* [23], which reduces the size of the vector $\hat{A}$ simply by setting to zero coefficients which do not contribute to the estimation of the output. Lasso estimates predictors’ coefficients by solving the following minimization problem [24]

$$\begin{array}{c} {\hat{A}}^{\text{lasso}}=\underset{a_{i}}{\text{argmin}}\sum_{i=1}^{n}(y_{i}-a_{0}-\sum_{j=1}^{m}x_{i,j}a_{j}{)}^{2} \end{array}$$ (4)

$$\begin{array}{c} \text{subject}\;\text{to}\;\sum_{j=1}^{m}\left|a_{j}\right|\leq \lambda \end{array}$$ (5)

where *y* and *x* are elements of *Y* and *X*, {*a*_*j*_} are the coefficients of ${\hat{A}}^{\text{lasso}}$ and *a*_0_ is the intercept. It is worth highlighting that λ is a free parameter that can be used to choose how much the lasso will shrink: for smaller values of λ more coefficients will be rounded to zero, hence eliminating the corresponding predictors from the model. A second method is the *stepwise selection* [24, 25]. This method determines a relevant subset of coefficients by starting from the intercept and adding predictors one-by-one, selecting those which maximize the model accuracy (with a process known as *forward stepwise*); then eliminates the predictors which contribute the least to the estimation of the output (the *backward stepwise*).

### 2.6 Two-dimensional interpolation

Interpolation is a type of estimation that aims to find new data points based on a finite set of known ones, typically by calculating intermediate values between two available points using a specific function. Two-dimensional interpolation methods are needed to interpolate points in a plane. A common technique is to recur first to triangulation to generate a 2D surface by connecting given points via triangles, respecting specific rules given by the triangulation technique. For example, the *Delunay* triangulation [26] creates a set of triangles with the property of not including any other point inside the circumscribed circle. When the value at an arbitrary point is queried, the 2D surface is first visited to find inside which triangle the point falls in and, second, interpolation algorithm such as nearest-neighbor [27], linear [28], cubic [29] is applied, using only points belonging to the selected triangle for the estimation.

## 3 Soft sensors modeling

This section presents a stochastic approach for designing different soft sensors capable of estimating climatic parameters for different purposes, following the hierarchy presented in Fig 5. For each type of soft sensors, we present the mathematical procedure used to obtain them and their application to estimate climatic condition within a greenhouse.

### 3.1 Regressed soft sensors

These sensors are models capable of estimating the value of a climatic variable in a given location in terms of readings from a set of sensors placed elsewhere by using coefficients obtained as results of a linear regression.

As subsection 2.5: Linear regression techniques explains, the key elements to perform regression are the *X* matrix and the *Y* vector. We distinguish the training phase, in which *X* and *Y* are given and the regression coefficients are computed, from the operational phase, in which *Y* is computed. In the training phase, the column vector $Y=[\begin{array}{cccc} y(t_{1}) & y(t_{2}) & \ldots & y(t_{n}) \end{array}{]}^{T}$ stores the measurements taken at time *t*_1_ … *t*_*n*_ by the sensor we want to model (defined as *target model*), while

$$\begin{array}{c} X=[\begin{array}{cccc} x_{1}\left(t_{1}\right) & x_{2}\left(t_{1}\right) & \cdots & x_{m}\left(t_{1}\right)\\ \vdots & \vdots & & \vdots \\ x_{1}\left(t_{n}\right) & x_{2}\left(t_{n}\right) & \cdots & x_{m}\left(t_{n}\right) \end{array}]=[\begin{array}{c} x(t_{1})\\ \vdots \\ x(t_{n}) \end{array}] \end{array}$$

is an *n* by *m* matrix collecting measurements taken at the same *n* instants from *m* other sensors (defined as *input sensors*). In general, inside the *X* matrix, any sensed data can be inserted, such as temperature, solar radiation, relative humidity, wind speed, and so forth. In fact, in the next step of the methodology, the *lasso* and *stepwise*, described in subsection 2.5: Linear regression techniques, automatically remove variables that do not contribute to, or may even reduce, regression accuracy. This step allows turning the *X* matrix into a smaller matrix, denoted as $\bar{X}$.

However, using data from input sensors is only sometimes sufficient to explain all phenomena that affect target models. While the model can represent some recurring events (such as the day-night cycle of air temperature) by observing persistent sensors (e.g., AT sensor or light sensor), other phenomena are not captured by persistent sensors, being “local” phenomena.

If local phenomena are time-dependent (e.g., shades), they can still be included in the model by adding to the input set some variables explicitly representing the time at which each measurement has been collected. This leads to a new matrix $\tilde{X}$ defined as $\tilde{X}=[\begin{array}{ccccccc} M & | & D & | & H & | & \bar{X} \end{array}]$ where *M*, *D* and *H* are binary maps defined as:

$$\begin{array}{cc} M & =[\begin{array}{ccc} m_{1}\left(t_{1}\right) & \cdots & m_{12}\left(t_{1}\right)\\ \cdots & & \\ m_{1}\left(t_{n}\right) & \cdots & m_{12}\left(t_{n}\right) \end{array}]\\ D & =[\begin{array}{ccc} d_{1}\left(t_{1}\right) & \cdots & d_{31}\left(t_{1}\right)\\ \cdots & & \\ d_{1}\left(t_{n}\right) & \cdots & d_{31}\left(t_{n}\right) \end{array}]\\ H & =[\begin{array}{ccc} h_{1}\left(t_{1}\right) & \cdots & h_{24}\left(t_{1}\right)\\ \cdots & & \\ h_{1}\left(t_{n}\right) & \cdots & h_{24}\left(t_{n}\right) \end{array}] \end{array}$$

*M*, *D*, and *H* stand for Month, Day, and Hour, respectively. The *i*-th row of *M*, *D*, and *H* encodes the timestamp of the samples at the *i*-th row of the matrix $\bar{X}$, such that only the entry that has column index matching the month, day and hour of the timestamp is set to one, while other the row elements are set to zero. For instance, if the *i*-th row of $\bar{X}$ has the timestamp 2021–10-15 13:00, then the *i*-th rows of *M*, *D* ad *H* will have a 1 in column index 10, 15 and 13, respectively:

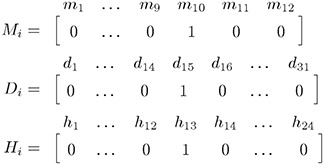

It is worth noting that such a procedure can capture local phenomena that should be included in the model and mitigate the effect of phenomena that affect only input sensors and thus should be excluded from the model to avoid altering the estimation process.

Having defined what the *Y* vector and the *X* matrix mean for *R-Soft Sensors* and which form they should have, it is now possible to give a more mathematical definition for such soft sensors. Assuming *S*_*k*_ to be a source of data (defined as *target*) placed in a point of interest that monitors the value of the variable *V*, then *S*_*k*_ can be described as the function

$$V(t)=S_{k}\left(t\right)$$ (6)

that for each instant of time *t* produces a value *V*(*t*). The corresponding *R-Soft Sensor* ${\hat{S}}_{k}$, which generates the estimate $\hat{V}\left(t\right)$ of *S*_*k*_, can then be described as

$$\begin{array}{c} \hat{V}\left(t\right)=\hat{S_{k}}\left(\tilde{x}t\right) \end{array}$$ (7)

$$\begin{array}{c} =\tilde{x}\left(t\right)\hat{A} \end{array}$$ (8)

$$\begin{array}{c} =\tilde{x}\left(t\right){\left({\tilde{X}}^{T}\tilde{X}\right)}^{-1}{\tilde{X}}^{T}Y \end{array}$$ (9)

Where $\tilde{x}\left(t\right)$ is a row related to the timestamp *t* of the matrix $\tilde{X}$ calculated before, and *Y* is the column vector described at the beginning of the section.

The following two sections present the three types of *R-Soft Sensors*.

### 3.2 *Sparse Soft Sensors* and *Forecast Sparse Sensors*

**Fig 6:**
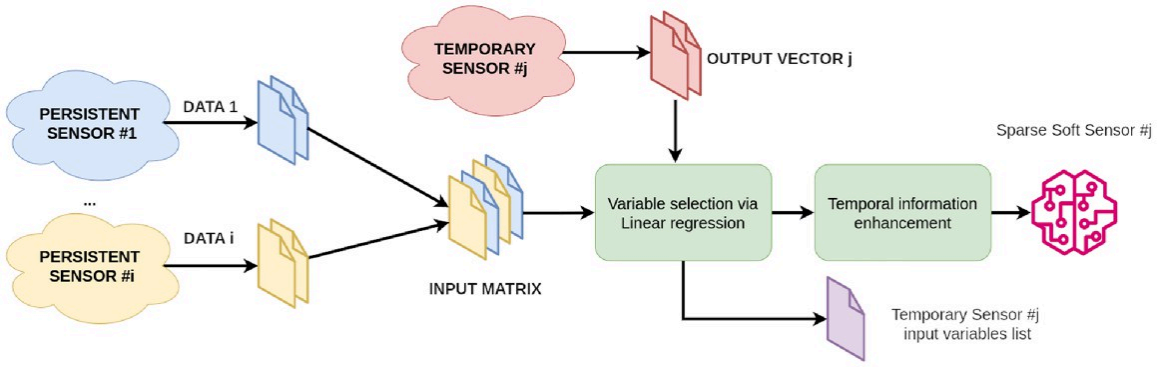
Creation flow of *Sparse Soft Sensors* starting from acquired data.

**Fig 7:**
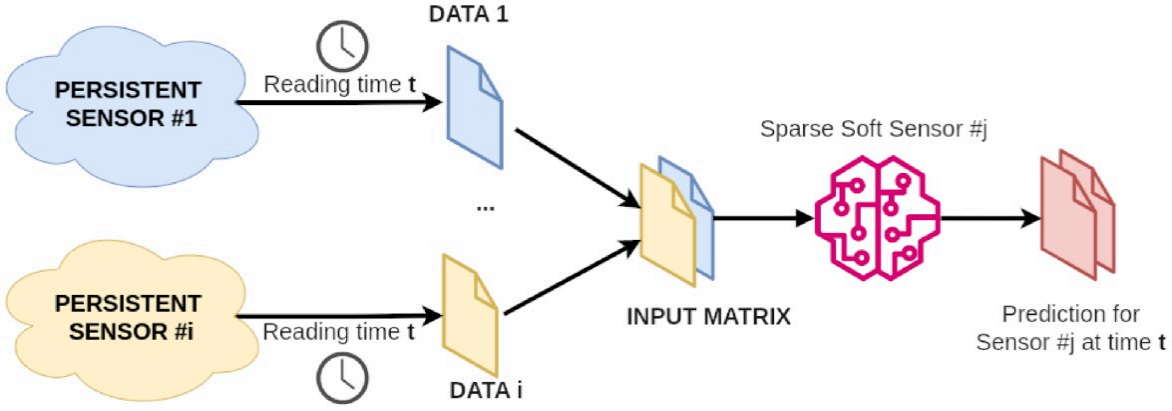
Runtime usare of *Sparse Soft Sensors* at time *t*.

In this paper, we define as *Sparse Soft Sensor* a special *R-Soft Sensor* where the matrix $\tilde{X}$ is generated from historical data gathered from all persistent sensors while the training vector *Y* consists of historical data coming from a single temporary sensor placed for a certain amount of time in a point of interest of the greenhouse. The temporary sensor plays the target role, and the corresponding *Sparse Soft Sensor* is the target model that keeps producing reliable data throughout time, even if the source of data is removed. Fig 6 shows the complete set of operations to create the model for a given temporary sensor placed in a point of interest starting from data collected from the set of fixed ones, whereas Fig 7 shows runtime operations to use such model to estimate the variable at time *t* in the corresponding point of interest.

*Sparse Soft Sensors* allow the modeling of greenhouse microclimate in real-time according to data obtained from persistent sensors. However, there are many cases in which the knowledge of the internal status of a greenhouse is not enough to choose which agricultural actions need to be undertaken. For instance, crop protection against pests or diseases needs information about the near future to determine which risks the crop can undergo and take action to avoid future damages. Typically, information about climatic conditions for the near future is provided by weather forecasts, which often refer to a wide geographical area. To model future values of climatic variables in specific points of interest of the greenhouse, we can slightly change the described regression methodology such that the *X* matrix is filled with the weather forecast data while the *Y* vector contains data read from persistent or temporary sensors depending on the point of interest; the readings should be aligned to the time instant weather forecast is referred to. This new type of soft sensor is defined as *Forecast Sparse Sensor*.

Weather forecast values typically have a lower time granularity than sensor readings; therefore, they cannot be directly put in relation in the regression operation. To solve this problem, sensor readings are averaged over the same time window forecasts refer to, so that both sensed and forecast values have the same time granularity, and Eq 9 can be used.

### 3.3 Anomaly detection soft sensors

Detection of anomalous readings from a sensor is essential to discover failures and avoid taking wrong agricultural actions. Furthermore, in our approach, anomalous readings from persistent sensors introduce noise in soft sensors based on them and thus their early detection is crucial.

**Fig 8:**
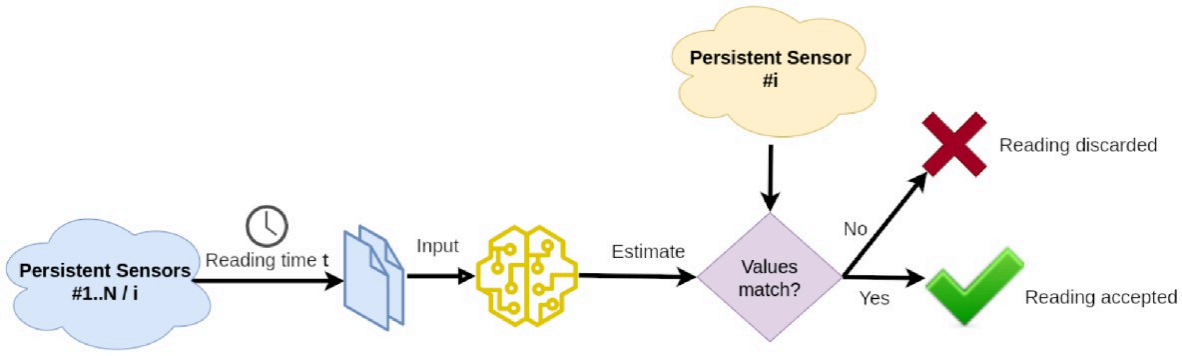
Runtime detection of anomalies. Runtime exploitation of models to estimate the correctness of data read from a persistent sensor.

To validate the measurements obtained from each persistent sensor, the proposed stochastic modeling was exploited again. For each persistent sensor, its historical data (*Y* vector) is put in relationship with those of the other persistent sensors (*X* matrix) to create a regression model, and thus a soft sensor defined as *Anomaly Detection Soft Sensor*. As explained in Fig 8 these soft sensors are used at runtime to estimate the climatic variable associated with each persistent sensor in terms of readings from the other ones. Then, the estimation is compared to the actual sensor reading and a difference is interpreted as an anomaly. It is worth noting that this mechanism can implement a reciprocal supervision of the persistent sensors and models can be continuously re-trained with new incoming data.

### 3.4 *2D-Sensor*

*Sparse Soft Sensors* and *Forecast Sparse Sensors* allow monitoring a fixed number of locations without needing physical permanent sensors. However, it might be interesting to monitor the same variables in arbitrary locations of the greenhouse area without introducing other temporary sensors and training new *Sparse Soft Sensors*. The 2D-interpolated estimation of a physical variable is given by Eq 10, which estimates the variable values ${\hat{V}}_{t}^{p_{x},p_{y}}$ for a given location (*p*_*x*_, *p*_*y*_) at time *t*. This type of soft sensor is defined as *2D Sensor*.

$${\hat{V}}_{t}^{p_{x},p_{y}}=S_{2D}(p_{x},p_{y},t)$$ (10)

The *2D-Sensor* takes three parameters as input, i.e., the spatial position of the point to monitor (*p*_*x*_, *p*_*y*_) and the time *t* for which the estimation has to be done. It is worth highlighting the presence of the time parameter: the interpolation process uses data from sensors (persistent or soft) to create a surface and compute the value for the desired position. However, since climatic variables evolve over time, it becomes crucial to explicitly choose the time the wished result will refer to, forcing the interpolation function to use input data obtained in that specific time moment.

The interpolation mechanism is flexible and can be combined with the previously described *R-Soft Sensors*. A *2D-Sensor* can create a microclimatic map referring to either the current time if it is fed by persistent sensors and *Sparse Soft Sensors* or a future time if it is fed by *Forecast Sparse Sensors*.

## 4 Results

This section will show results for the case study presented in section 2: Material and methods, where microclimatic data have been acquired from nine sensors located between two adjacent greenhouses. The experimental setup has been replicated in two different farms to show the portability of the methodology. In particular, we:

- create a microclimatic map by using the methodology described in section 3: Soft Sensors modeling;
- model *Forecast Sparse Sensors* by using weather forecast as input to forecast temperature in the various points of interest instead of predicting current temperature values;
- design an *Anomaly Detection Soft Sensor* for each persistent sensor for fault detection purposes.

### 4.1 *Sparse Soft Sensors* design and validation

Data from temporary sensors were acquired for about eleven months, from late August 2021 to early June 2022, and used to create nine vectors *Y*_*k*_ to apply the methodology previously described. The input matrix *X* collected data from the Evja microclimatic station and the weather station, such as internal and external air temperature, relative humidity, solar radiation, air pressure, wind speed, and also more complex indexes such as dew point and wet-bulb temperature, for a total of 24 different input variables and more than 14, 000 readings over the observation period. Furthermore, in each row, we collected the measurements of the three previous time instants (hence up to 45 minutes in the past) from the weather station and Evja station

$$\begin{array}{c} X=[\begin{array}{ccccccccc} x_{1}\left(t_{3}\right) & \cdots & x_{m}\left(t_{3}\right) & x_{1}\left(t_{2}\right) & \cdots & x_{m}\left(t_{2}\right) & x_{1}\left(t_{1}\right) & \cdots & x_{m}\left(t_{1}\right)\\ \vdots & & \vdots & & & & & & \vdots \\ x_{1}\left(t_{n}\right) & \cdots & x_{m}\left(t_{n}-1\right) & x_{1}\left(t_{n}-1\right) & \cdots & x_{m}\left(t_{n}-1\right) & x_{1}\left(t_{n}-2\right) & \cdots & x_{m}\left(t_{n}-2\right) \end{array}] \end{array}$$

bringing the total input matrix’s size up to 72 columns.

A MATLAB script was created to perform the regression procedure automatically. The script first takes the *X* matrix and the *Y* vectors and splits them into two parts, one constituted by 80% (first 40% and last 40%) of the total measurements to train the model and the remaining 20% to test it. The script then proceeds by using MATLAB’s stepwiselm function, which computes a stepwise regression by applying a forward selection in sequence to add all needed variables to the model and a backward selection to eliminate previously added but not statistically significant. Before computing the regression, the input matrix *X* and output vector *Y* have been normalized to feature zero mean and unitary variance. In our case, the selection criterion was set to adjrsquared, meaning that the function will focus on obtaining the highest Adjusted-*R*^2^ value by adding input variables to the current set as long as they increase it by at least 0.0001 (the set threshold). After this operation, the matrix was enhanced with temporal information as described in subsection 3.1: Regressed Soft Sensors; the resulting $\tilde{X}$ matrix is used in a final regression process to obtain the coefficients of the models representing the *Sparse Soft Sensors*.

The following three evaluation metrics have been used:

- adjusted-R-squared
  $$\begin{array}{c} R_{\text{Adj}}^{2}=1-\frac{n-1}{n-k-1}\frac{\sum_{i=1}^{n}(y_{i}-{\hat{y}}_{i}{)}^{2}}{\sum_{1}^{n}{\left(y_{i}-\bar{y}\right)}^{2}}, \end{array}$$
- Adjusted Root Mean Square
  (RMSE_Adj_), calculated as the ratio between the Root Mean Square Error (RMSE) and the Root Mean Square (RMS) of *y*
  $$\begin{array}{c} {\text{RMSE}}_{\text{Adj}}=\frac{\text{RMSE}(Y,\hat{Y})}{\text{RMS}(Y)}=\sqrt{\frac{\sum_{i=1}^{n}{\left(y_{i}-{\hat{y}}_{i}\right)}^{2}}{\sum_{i=1}^{n}y_{i}^{2}}}; \end{array}$$
- Relative Root Squared Error (RRSE), calculated as the ratio between the Root Mean Square Error the standard deviation *σ*
  $$\begin{array}{c} \text{RRSE}=\frac{\text{RMSE}(Y,\hat{Y})}{\sigma (Y)}=\sqrt{\frac{\sum_{i=1}^{n}{\left(y_{i}-{\hat{y}}_{i}\right)}^{2}}{\sum_{i=1}^{n}{\left(y_{i}-\bar{y}\right)}^{2}}}. \end{array}$$

Where *n* is the number of samples, *k* the row number of coefficient vector *A*, *y*_*i*_ and ${\hat{y}}_{i}$ are elements belonging respectively to vectors *Y* and $\hat{Y}$, and $\bar{y}$ is vector *Y*’s mean value.

In S1 and S2 Tables results for all sensors of both farms have been reported. Table 1 lists the metrics for air temperature and humidity for the two points of interest further away from the Evja station (number 9 in Fig 4).

**Table 1:** Accuracy results (R-squared, Adjusted Root Mean Square Error, and Relative Root Squared Error) for air temperature and RH models relative to the farthest point of interest from the Evja station in both farms.

Sensor		$R_{Adj}^{2}$	Adj RMSE	RRSE
First Farm	Temperature	0.99	0.03	0.11
	RH	0.94	0.05	0.24
Second Farm	Temperature	0.87	0.10	0.30
	RH	0.94	0.04	0.21

**Fig 9:**
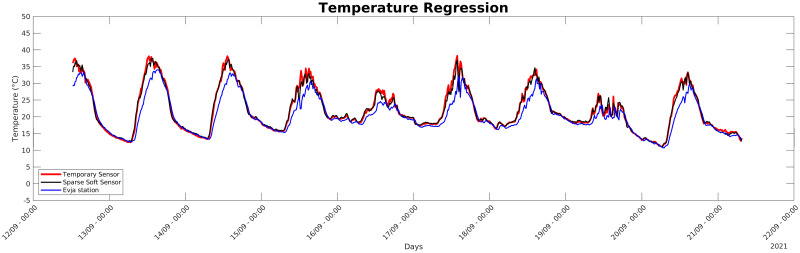
Validation phase. Plot for validation phase of regression for sensor #9.

As it is possible to see in those tables, each model has a precision better than 85% both for training and testing and most reach the accuracy of 98–99%, meaning that *Sparse Soft Sensors* can estimate very precisely temperatures at the given points in space. The time series for the temperature *Sparse Soft Sensor* of the First Farm of Table 1 is reported in Fig 9 and compared to the actual value of the temporary sensor used for training and to the values provided by the Evja station. Estimated values follow the actual ones as desired, whereas the behavior of the Evja station serves as proof of the heterogeneity of the greenhouse’ microclimate and of the potentiality of our methodology that can transform inaccurate readings of a single central sensor into much more accurate local information.

### 4.2 Impact of temporal information on the model

In order to test how much the time information impacts the regression model, two tests were conducted. The first one called the *Unmodified-output test*, aimed to check the difference between a standard regression model obtained from a given set of data and a regression model obtained by adding temporal information to available inputs (timed model). The second test, labeled the *Modified-output test*, repeats what was done in the previous one, but outputs were altered in order to introduce a local daily event, by forcing a temperature decrease of 5°C only between the 16:00 and 18:00 every day. By doing this an important recurring event, although synthetic, was introduced in the output dataset, to see how well-timed and un-timed regression models were capable of handling it.

**Fig 10:**
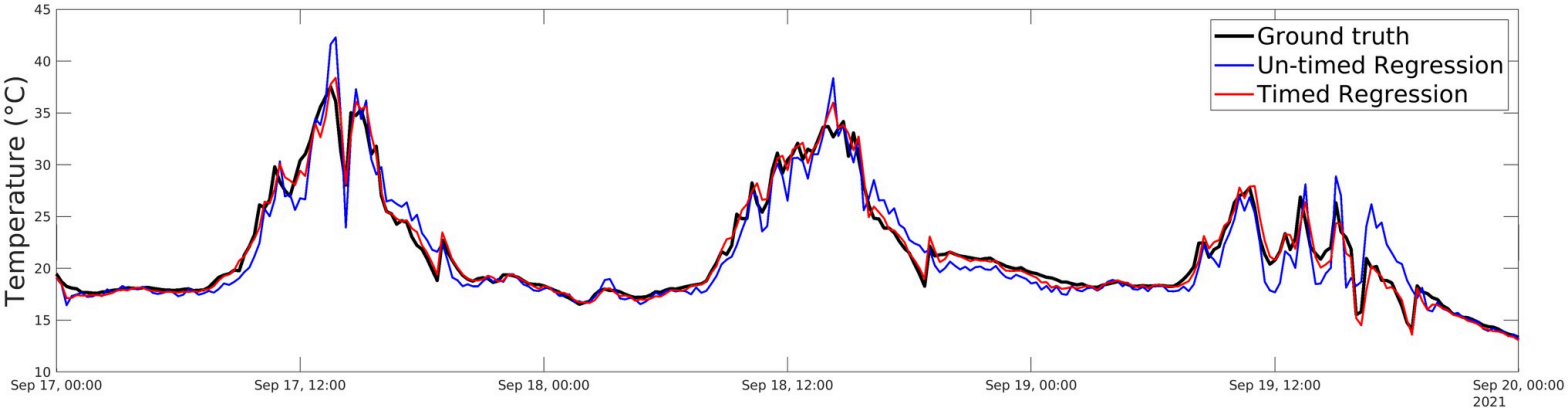
Comparison between timed and un-timed models. Accuracy comparison for un-timed and timed models when a local recurring event occurs.

Table 2 reports the values of the accuracy metrics for each test and each model. In the case named “Modified-output”, the timed model is more accurate since it allows for coping with the variation introduced by the presence of the local phenomenon, which cannot be detected by persistent sensors. However, even for the Unmodified-output, the timed model performs better than the un-timed one with an average accuracy increase of 2% (0.99 against 0.97). Such difference can be explained by the presence of local phenomena which may also affect the persistent sensors and which the timed model still manages to capture, differently from the un-timed one, thus resulting in an increase in accuracy. Fig 10 compares the behavior of the two models in the Modified-output case: the timed model (red line) is much closer to real values (black line) with respect to the un-timed one (blue line), with a difference in accuracy of 4%.

**Table 2:** Metrics for each model and test. The timed model is always more accurate than the un-timed one.

Output	Metric	Timed Model	Un-timed model
Unmodified-output	$R_{Adj}^{2}$	0.99	0.97
	Adj RMSE	0.03	0.04
	RRSE	0.11	0.16
Modified-output	$R_{Adj}^{2}$	0.99	0.95
	Adj RMSE	0.07	0.07
	RRSE	0.21	0.21

### 4.3 Integration of weather forecast

The process described in subsection 3.2: *Sparse Soft Sensors and Forecast Sparse Sensors* has been used to establish a relationship between the weather forecasts and the temperature values of the weather station with the aim of obtaining temperature forecasts for this specific location. The weather forecasts for the geographical zone were retrieved from a specialized website, providing an estimation of temperature, air pressure, and relative humidity for each hour of the day. Such measurements have been assumed as average values for the one-hour span the forecast refers to. Therefore, we applied the same assumption to the sensed values used in the *Y* vector for the regression, i.e., each value was the average between readings from thirty minutes before up to thirty minutes after the time stamp of the corresponding forecast value. For example, if a forecast refers to 10:00 am, the corresponding value in the *Y* vector was computed as the average between the sensors’ readings from 9:30 am to 10:30 am.

**Fig 11:**
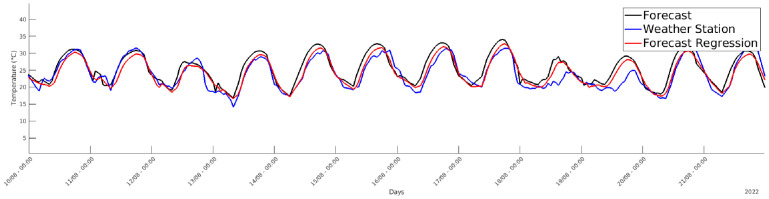
Validation of forecast model. Validation data for the forecast model against the original forecast and the values sensed by the weather station.

Regression results show that model accuracy reached an R-squared value greater than 0.8 for both the calibration and validation phases. Fig 11 shows the real temperature measurements (in blue) of the weather station near the greenhouse, the temperature forecast of the day before relative to a nearby town (in black), and the corresponding predicted greenhouse temperature forecast (in red). The obtained model provides a good estimation of future conditions near the greenhouse for most of the time. However, it is worth noting that such results were obtained by using a free weather forecast web service hence with limited accuracy, meaning that a better forecast service can easily surpass the reported performance.

### 4.4 2D map generation and validation

To further increase the area covered by *2D-Sensor*, six more temporary sensors were placed; they are denoted by letters in Fig 4. Such sensors were installed in intermediate positions between the ones already present, to avoid having too much distance between two modeled sensors.

After modeling fourteen temperature points of interest as *Sparse Soft Sensors*, the area delimited by them was split into a grid whose points were used to design the 2D map. The *2D-Sensor* was implemented by using the griddata interpolation function of MATLAB fed by positions and estimated values of all the *Sparse Soft Sensors*. This function is called every time a new set of measurements is available for the *Sparse Soft Sensors* (i.e., when new samples from the persistent sensors are available).

**Fig 12:**
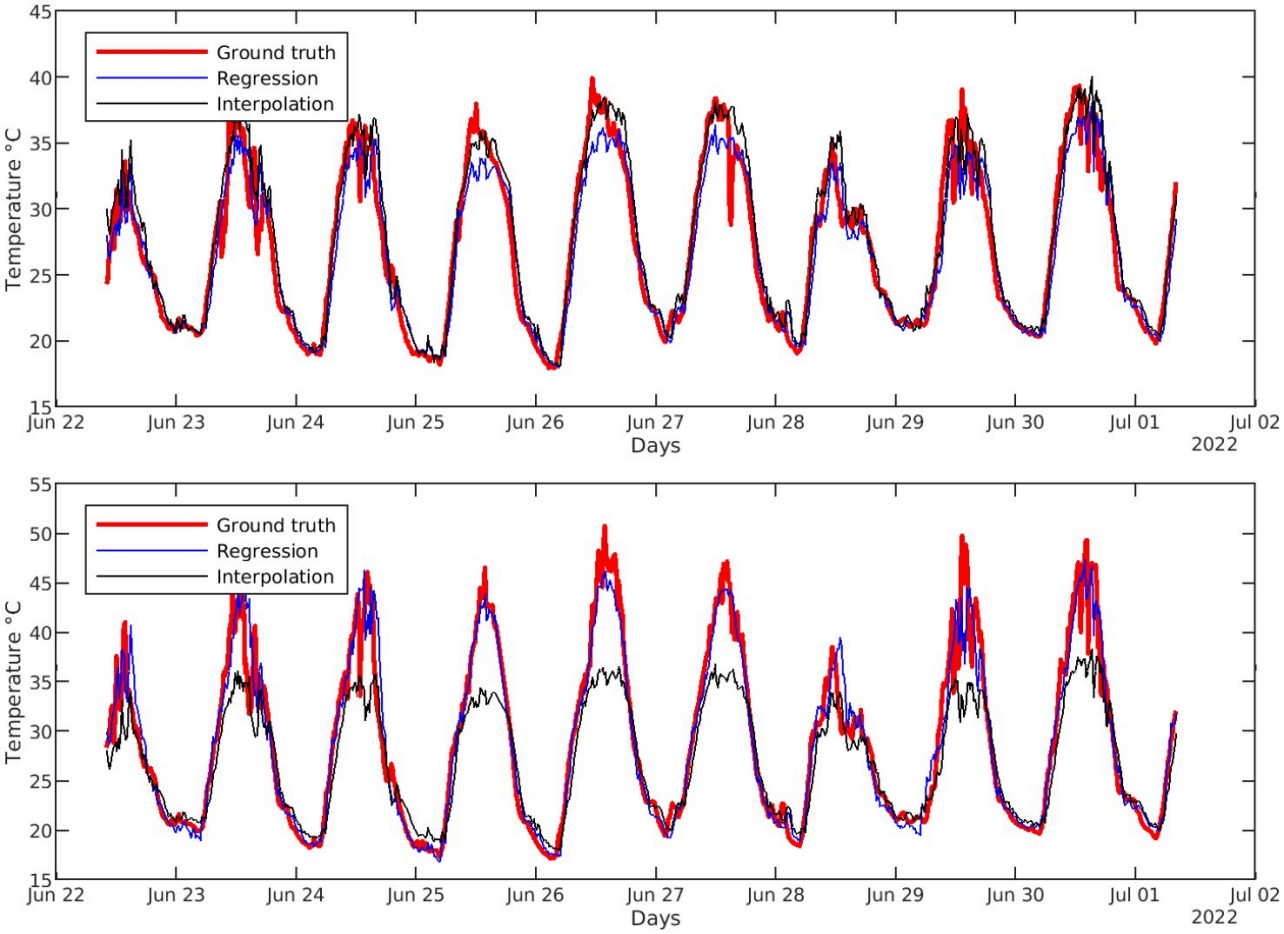
Comparison between *Sparse Soft Sensors* and *2D-Sensor* for two positions. Comparison between estimated values with *Sparse Soft Sensors* and interpolation for two locations in the map. The first plot refers to a central location where interpolation estimates temperature precisely, while the second one refers to a corner of the working area where interpolation is less accurate than *Sparse Soft Sensors*.

**Fig 13:**
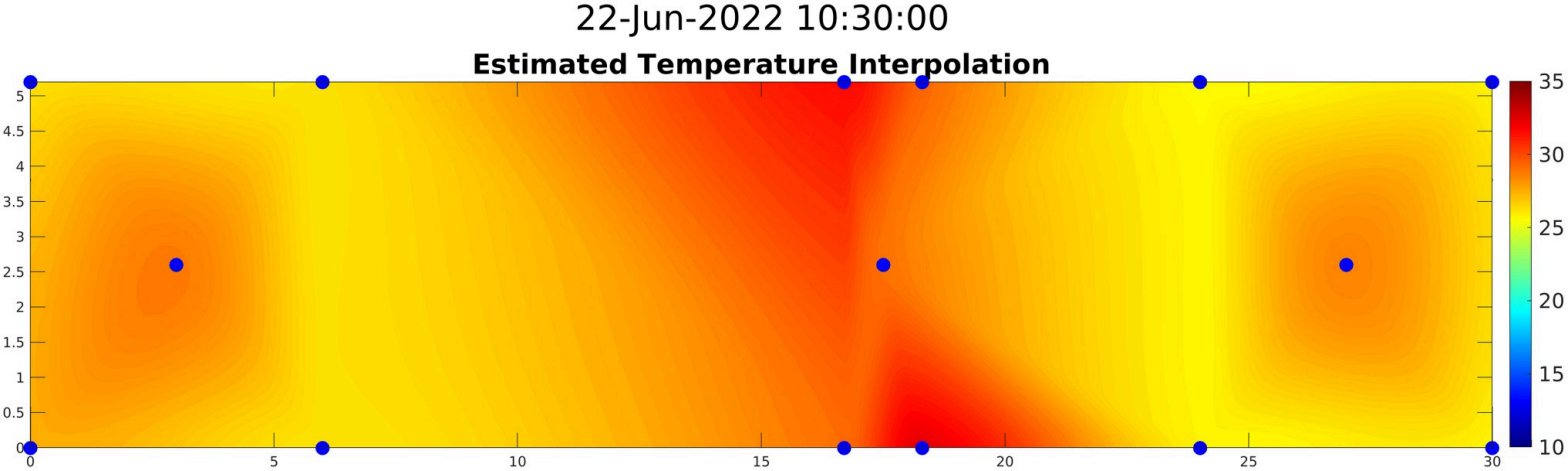
Temperature map obtained by combining estimation from *Sparse Soft Sensors* and the *2D-Sensor*.

The validation of the interpolation procedure was done by comparing, for each of the fourteen monitored spots, the actual value (obtained with a real sensor), the value from the corresponding *Sparse Soft Sensor*, and the *2D-Sensor*’s estimation obtained through interpolation of all the *Sparse Soft Sensors* except the examined one. Fig 12 shows the values for a central location (upper plot) and for a corner location (bottom plot). Results show that the difference between the *Sparse Soft Sensors* and the interpolated values increases for the points closer to the border of the examined area; in some points at the center of the area, the interpolation is even more accurate than the *Sparse Soft Sensors*. We can conclude, then, that *Sparse Soft Sensors* are necessary for peripheral points of interest, while for central points of interest the *2D-Sensor* is enough, and the creation of specific *Sparse Soft Sensors* is not needed. Fig 13 gives instead an idea of how the final result of the interpolation process should look like, basically creating a heat map of the monitored area to let farmers know at first glance how the situation inside the greenhouse is.

### 4.5 Validation of anomaly detection

**Fig 14:**
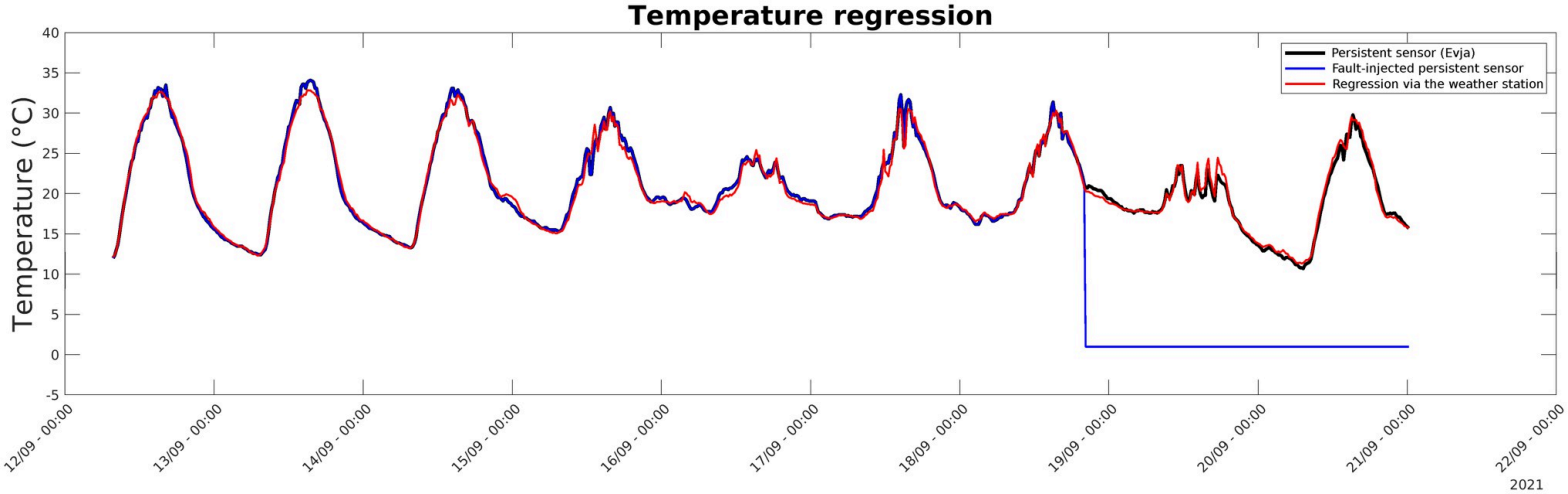
Comparison between the faulted sensors and model’s predicted values. Comparison between temperature readings from the Evja sensor and the same variable modeled through the weather station. The regression model detects the fault injected in the sensor.

To validate the anomaly detection methodology explained in subsection 3.3: Anomaly Detection Soft Sensors, a model of each Evja sensor was built based on data coming from the weather station and vice versa. Results showed that for physical variables that were monitored by both stations, the prediction accuracy was very high (*R*^2^ > 0.8), whereas the prediction of variables not sensed in the other station (e.g., the soil moisture not present in the weather station) was imprecise. Fig 14 shows the original behavior of the Evja temperature sensor, its fault-injected version, and the predicted behavior by using data from the weather station. The anomalous behavior of the sensor is denoted by the difference between its output and the corresponding prediction. Therefore, it is possible to continuously check the validity of a data source simply by using incoming data from the other one.

## 5 Discussion

We are proposing an approach to predict the value of a climatic variable in the greenhouse at a given time and space location by using available data for that time either obtained from real sensors, in case of current time, or from weather forecast in case of future time. For each climatic variable of interest, its behavior as a function of the space is modeled either by correlating it to that of temporary sensors previously placed in the same location or by interpolating known values in the neighborhood. The introduction of temporal information as input to the training phase further enhances accuracy by modeling recurring daily or monthly phenomena that locally affect the specific point of interest.

**Fig 15:**
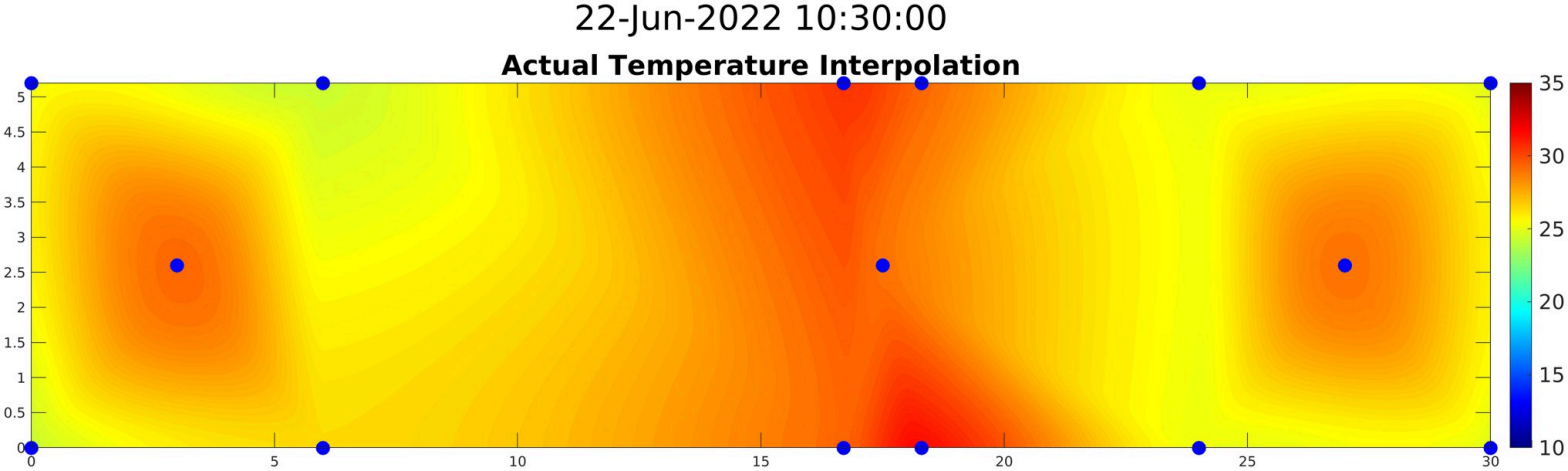
Greenhouse heat map. 2D temperature map of the greenhouse obtained by interpolating temperature values sensed in specific positions (blue dots).

As shown in Fig 15, there could be up to five Celsius degrees of difference in temperature between two points of the greenhouse used for our experiments; according to well-known disease prediction models [11], such temperature differences may lead, over time, to significantly different probabilities of new infection. Therefore, the proposed approach, by going beyond the homogeneity assumption for climatic variables, allows to implicitly increase the effectiveness of state-of-art agronomic prediction models. In the future, it enables the adoption of space-customized use of fertilizers and pesticides, as expected by the development of agricultural robots [30].

We adopted the concept of soft sensors, i.e., predicted data for a given point of interest that behaves as a sort of virtual sensor placed in that point. This concept has the following advantages:

- Affordability: the number of actual network-connected sensors can be kept small, while less expensive sensors (a.k.a. data loggers) can be used to acquire data needed for training. Such sensors can be re-used in different positions to extend monitored points without any extra cost. In some specific cases, a very good forecast service and a high quality monitoring station (e.g., that provided by public authorities for environmental monitoring) are enough to put in place the micro-climatic mapping of the greenhouse.
- Scalability: it is possible to start with a small number of *Sparse Soft Sensors* in specific points of interest (e.g., close to a certain group of plants), then recur to a *2D-Sensor* to obtain a rough estimation of climatic variables in many other points and, finally, re-iterate training to create new *Sparse Soft Sensors* if more accuracy is needed in new points of interest (as shown in Fig 13).
- Flexibility: the same algorithm can be used for different purposes, i.e., local estimation, forecasting and anomaly detection; according to Fig 5 and Eq 10 a *2D-Sensor* is independent of the variable that feeds it, e.g., current data or forecast; finally, the set of temporary but static sensors could be replaced by an array of sensors aboard a fleet of vehicles periodically moving in the greenhouse to perform agricultural operations.
- Architectural neutrality: a software tool encapsulating the prediction methodology can be alternatively hosted in the cloud, on a machine in the farm, and even inside a smarter sensor.
- Compatibility with agricultural operations: the use of virtual sensors is compatible with mechanical operations in the greenhouse which sometimes interfere with sensor cases and cabling.

The main objective of a prediction methodology is to capture the rules that describe the behavior of a system (in this case the greenhouse microclimate). A *model-driven* prediction approach relies on the description of the physical and structural rules at the basis of an observed behavior. Even if the results can be very good, it usually requires a high expertise in system description (e.g., knowledge of complex physical laws and features of involved materials). Vice versa, a *data-driven* prediction approach aims at capturing cause-effect relationships by correlating data produced by the same causes. It always relies on sensing and data analysis, e.g., by using regression techniques or neural networks. The proposed prediction approach is fully data-driven and is based on linear regression. In the agricultural context, a data-driven approach shows some important advantages over the model-driven approaches present in the literature:

- it is easier and cheaper since it does not rely on expert people who build the physical model of the environment;
- it can be “re-executed as is” for several different environments, e.g., for different kinds of greenhouses, for a tunnel greenhouse which becomes open field in certain periods of the year, for the same greenhouse in which a different kind of crop changes the fluid dynamical characteristics of the space (e.g., because of a different height of the plants); this feature allows to freeze the methodology in a software tool to be used many times together with a set of temporary sensors to be placed for a given period of time and then moved.

Among the various data-driven approaches, linear regression (the one used in the proposed methodology) allows the creation of a “white box” prediction model in which it is possible to see which are the input variables that mostly affect the prediction output. We can say that it is intrinsically more *explainable* than a deep learning technique. According to literature, explainability is a desired feature for agronomic tools [31]. Furthermore, the training phase in regression techniques is usually less computationally expensive than for neural networks, which often recur to special GPU-based hardware architectures and consume more energy. Therefore, the computer requirements of the proposed approach better match the agricultural domain.

Another important discussion point is related to the scheduling of the training phase, in which the *Sparse Soft Sensors* are created, with respect to the operational phase in which they are used for prediction. Ideally, the training phase should use data collected over a time span of one year to capture all the seasonal phenomena that affect the climatic behavior. However, if climatic conditions are quite stable over the year, it is possible to train over just a few months, but clearly, a model trained on winter data hardly predicts accurately in summer. It is worth noting that the shape of the plants may change air circulation and introduce shades, and therefore the model of an empty greenhouse is usually different from that of a greenhouse full of plants. Usually, horticultural production in the greenhouse consists of a cycle of different kinds of crops. In this case, an interesting possibility consists of training a model for each kind of crop just for the time between sowing and harvesting. If this kind of cultivation is repeated every year in the same period, a software tool can create for the farmer a “library” of models, each of which can be reused in the following years.

## 6 Conclusion

Greenhouse internal microclimatic conditions frequently show high spatial variability that is frequently neglected in greenhouse models, preferring to assume climatic variables to be uniform. Overcoming this gap can contribute to optimizing greenhouse internal environmental conditions to enhance crop yields and quality and to optimize energy, water, and pesticide application.

This paper proposes a cost-effective methodology to allow farmers to monitor greenhouse climatic variables at different points of interest and to gradually extend to all greenhouse points to create a complete microclimatic map. A data-driven machine-learning approach based on linear regression has been developed to keep the computational effort low. Furthermore, linear regression allows better explainability for the model, giving higher visibility to data components on which inference is performed on. Indeed, the model can be fed directly with data from sensors, weather forecasts, and time information to reduce costs, especially when compared to a fixed sensing infrastructure. Moreover, the proposed approach also exploits inference to detect anomalies in the physical sensors, an essential functionality in an actual agricultural setup. The presented results demonstrate that the greenhouse internal temperature estimation at different points is more accurate than assuming a homogeneous temperature for the whole greenhouse, managing to reach a prediction accuracy of 99% for temperature and 94% for relative humidity; furthermore, false sensor readings are promptly detected. Finally, although estimation errors are inevitable, they are balanced by the possibility of reaching a spatial granularity that is impossible to achieve with real devices, thus allowing more precise agronomic operations that reduce production costs and increase production sustainability.

Possible development for this research includes exploring other machine-learning methods to achieve robustness in case of missing values, as well as exploiting forms of data collection, such as using mobile sensors on agricultural robots and drones in place of temporary sensors, taking advantage of their ability to reach multiple locations while performing their tasks.

## Supporting information

S1 Table First farm results. Accuracy results for each sensor in the first farm. For each sensor the first row contains calibration results, while the second row contains validation results.
(TIF)

S2 Table Second farm results. Accuracy results (R-squared, Adjusted Root Mean Square Error and Relative Root Squared Error) for each sensor in the second farm. For each sensor the first row contains calibration results, while the second row contains validation results.
(TIF)
