# Supplementary figures and images for: A spatio-temporal methodology for greenhouse microclimatic mapping
Source: PLoS One. 2024 Sep 19;19(9):e0310454. doi: 10.1371/journal.pone.0310454 (PMC11412499; doi:10.1371/journal.pone.0310454)

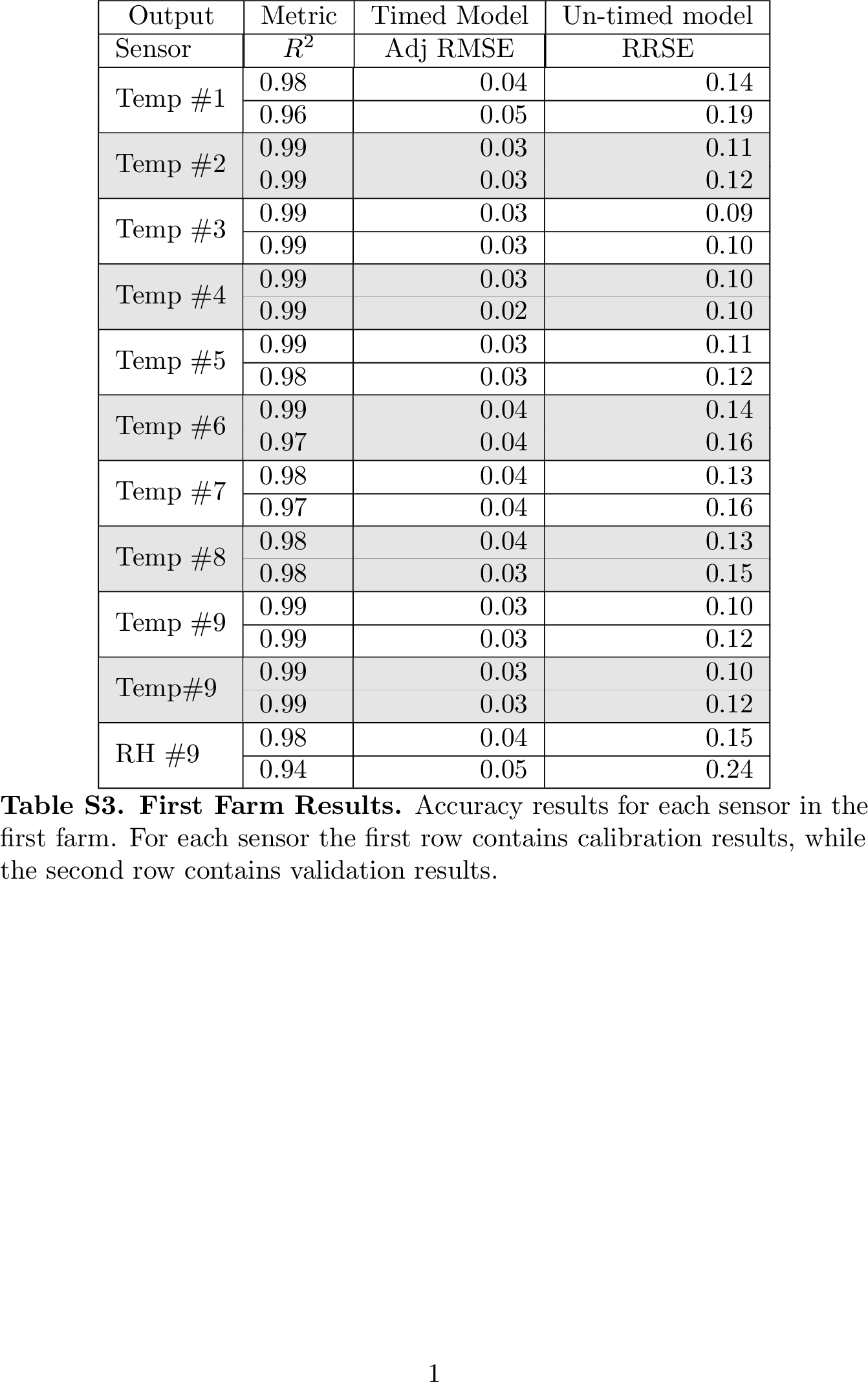

Supplement: S1 Table — Accuracy results for each sensor in the first farm. For each sensor the first row contains calibration results, while the second row contains validation results. (TIF) [file pone.0310454.s001.tif]

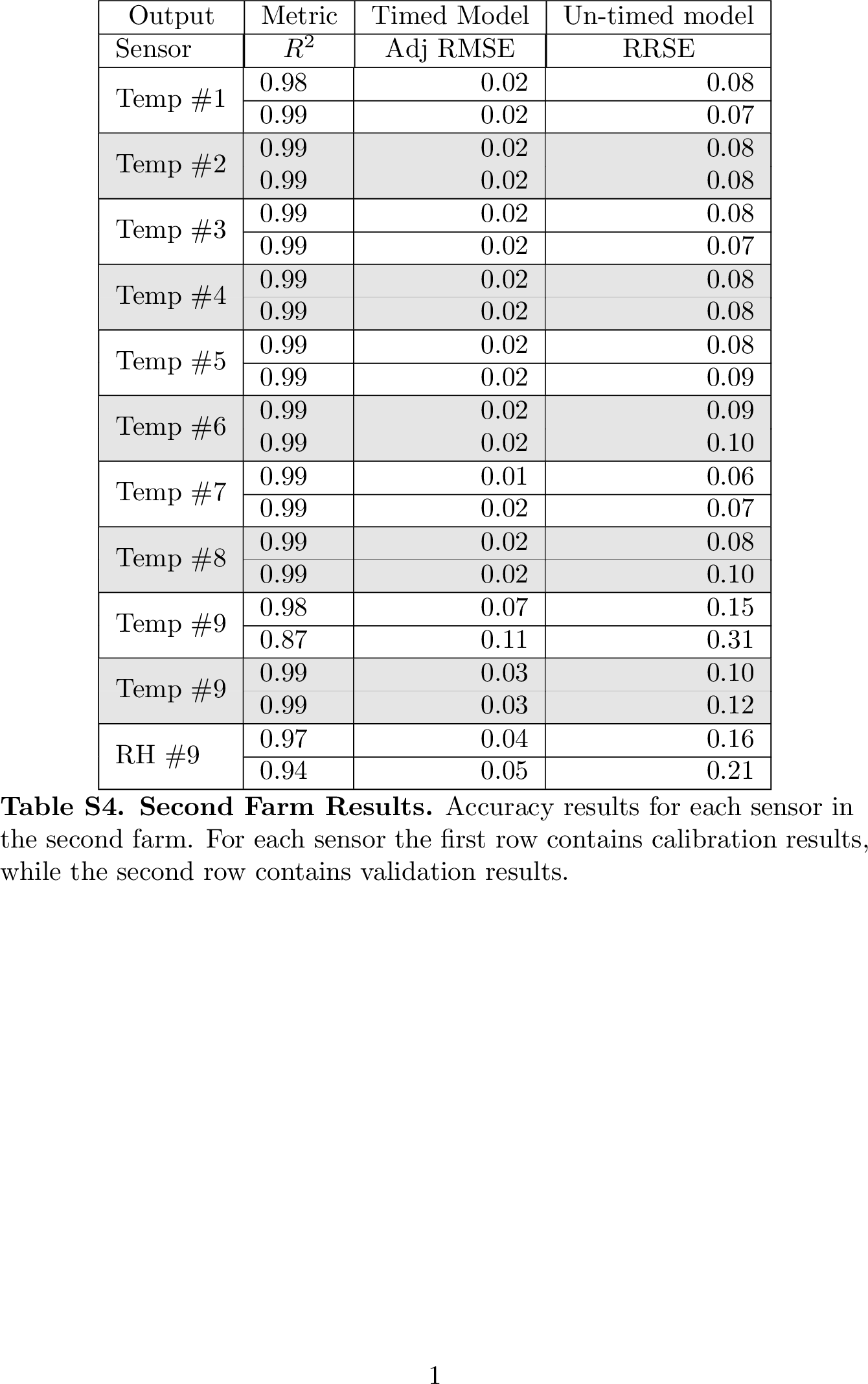

Supplement: S2 Table — Accuracy results (R-squared, Adjusted Root Mean Square Error and Relative Root Squared Error) for each sensor in the second farm. For each sensor the first row contains calibration results, while the second row contains validation results. (TIF) [file pone.0310454.s002.tif]
